# Determinants of Fruit Tree Adoption as a Climate Change Adaptation Strategy Amongst Smallholder Farmers in Lake Kyoga Basin: A Case Study of Budaka District, Eastern Uganda

**DOI:** 10.1155/tswj/9642641

**Published:** 2025-08-05

**Authors:** Nabalegwa M. Wambede, Kiconco Milliam, Ewongu Denis, Mulabbi Andrew, Tweheyo Robert, Mukisa Geoffrey

**Affiliations:** ^1^Department of Geography, Kyambogo University, Kampala, Uganda; ^2^Department of Sociology and Social Administration, Kyambogo University, Kampala, Uganda; ^3^Department of Media and Curriculum Studies, Muni University, Arua, Uganda

**Keywords:** adaptation, adoption, Eastern Uganda, farmers, fruit trees, perception, socioeconomic factors

## Abstract

This study investigated the socioeconomic determinants of fruit tree adoption amongst smallholder farmers in Budaka District, Eastern Uganda. Specific objectives included describing the characteristics of fruit tree gardens, mapping their spatial distribution, and analysing socioeconomic factors influencing adoption. This study is one of the first empirical studies in agroforestry to relate socioeconomic factors in Eastern Uganda to the spatial distribution of fruit trees. The study employed a combined approach incorporating GIS-based spatial mapping and socioeconomic analysis. A cross-sectional design was employed, with data collected from 276 randomly selected farmers, key informants, and focus groups. GIS was used to visualise the spatial patterns and descriptive statistics, and chi-square tests were applied to identify differences between adopters and nonadopters. Results indicated that fruit farming is predominantly undertaken by males aged 40 and above. Fruit tree distribution is concentrated in the north and northwest, grown on small holdings averaging 0.5 acres with 10–40 trees. Chi-square tests confirmed significant differences in age, labour type, farm size, and income between adopters and nonadopters, whilst there were no significant differences in gender, family size, and access to credit. Policy interventions should expand youth- and gender-inclusive extension services that support climate resilience and sustainable fruit tree farming, and address land tenure limitations to increase adoption.

## 1. Introduction

Plant cultivation emerged in the tropical regions of Central and South America, and Africa around 11,000–7000 years ago, driven by population growth and the shift towards sedentary lifestyles [1–6]. Domestication efforts were often driven by the desire to improve quality and yields, but the process experienced uncertainties and difficulties. Over time, new techniques for fruit tree domestication were developed based on factors like ease of vegetative propagation, as seen in species such as olives, dates, figs, and citrus, especially in the Mediterranean environments. Similar efforts also occurred in Sub-Saharan Africa, where domestication and integration of species like tamarind, shea, baobab, and later introduced species like mangoes and jackfruit provide both food security and livelihood [7, 8]. Techniques like self-fertilisation, bitterness reduction, and grafting accelerated the domestication process. Despite this history, fruit trees are still not widely used in many contemporary farming systems, particularly in Sub-Saharan Africa, because of limited institutional support, land scarcity, and economic barriers [9, 10].

Climate change, with its associated effects, continues to affect many communities and ecosystems globally. Extreme weather events like floods and droughts are increasingly being felt, culminating in adverse effects on community livelihoods. In Uganda, studies have confirmed that climate change phenomena are affecting many sectors of the economy and have even been reported as an obstacle to the attainment of Uganda's Vision 2040. The most affected sectors include agriculture and livestock, water, energy, human settlements, and transport [11]. The Lake Kyoga basin in Eastern Uganda is reported as one of the most affected regions, registering frequent droughts, floods, and landslides. Crop failures due to drought are a common feature in the region, leading some farmers to abandon farming due to the unpredictable rainfall patterns. Research by Obubu et al. [11] and Mubiru et al. [12] has reported changes in temperature and rainfall over the region, hence confirming that climate change is affecting the region. Lake Kyoga's vulnerability stems from low resilience levels, especially amongst subsistence farmers. Therefore, changes to the patterns and seasons of rainfall are swiftly felt in this area, pushing the farmers and authorities to forge adaptation strategies like agroforestry (fruit trees) that enhance resilience through livelihood diversification and help to mitigate climate change.

Agroforestry adoption has surged globally, especially in developing countries [13]. Fruit tree cultivation aligns with the goal of reducing poverty and hunger (MDG 1), but climate change poses a significant threat to these efforts [14]. Tropical regions boast a vast array of fruit trees, with over 1200 species in Africa alone. Adaptation is crucial in South Asia and Africa, where climate change has led to growing food insecurity [15]. Whilst fruit trees offer resilience due to their perennial nature, they are not immune to climate change impacts [16, 17]. Climate plays a major role in deciding perennial fruit crops' distribution, phenology, fruit quality, and diseases and pests' incidence [16]. The physiological and yield attributes of fruits are affected by changing global climate despite the increasing CO_2_ and temperatures that are needed by the plants for photosynthetic activity [18–23].

Fruit production is a prevalent activity in tropical regions but is underrepresented in climate change and adaptation studies [24]. Consequently, research on fruit tree adoption as a climate change adaptation strategy should be scaled up. This is because, although climate change affects fruit trees, fruit trees require CO2 for photosynthesis, where it is absorbed from the atmosphere and assimilated into their cellulose, lowering the atmospheric buildup. Fruit trees also contribute to a sustainable environment by cleaning the soil, cooling the cities, absorbing air pollutants, providing food, promoting plant diversity, and producing oxygen [8, 25–28]. The absorbed carbon is stored both above and below the ground. Despite the environmental benefits the fruit trees offer, their adoption into different agricultural systems, including agroforestry systems, is generally slow when compared with high-yielding cash crops like rice and corn. The limited understanding of factors influencing fruit tree adoption has hindered efforts to promote this potentially adaptive strategy [29].

The cultivation of fruit trees in a given area is contingent upon the presence of specific environmental conditions, including those of relief, climate, and soil. Places with reliable rainfall, rich and well-drained soils, and hot temperatures are more suited for fruit tree growing [7]. The occurrence of two different rainfall seasons in tropical and subtropical regions and the presence of reliable water sources such as rivers, lakes, aquifers, and wetlands contribute to agroclimatic diversity. This diversity enables the cultivation of a wide range of fruit trees, including mangoes, citrus, guavas, papayas, and jackfruits. As a result, environmental conduciveness remains a significant parameter influencing the geographical distribution and productivity of fruit tree systems worldwide [30].

Fruit production constitutes an essential component of horticulture, playing a pivotal role in ensuring food and nutritional security at the community level and in maintaining environmental hygiene [31]. The cultivation of fruit trees is of significant importance in the process of ‘maximising plant water availability by maximising the infiltration of rainfall; minimising unproductive water losses (evaporation, deep percolation, and surface run-off); increasing soil-water holding capacity; and maximising root depth' [32–34]. Additionally, fruit trees serve to safeguard wildlife and enhance the aesthetic appeal of their surroundings. Nevertheless, irrespective of their economic value, fruit trees have historically constituted a vital source of sustenance for humans and domestic animals alike. Consequently, a greater number of fruit species were cultivated in the past than is the case today [5].

The acceptance of fruit trees amongst small-scale farmers is largely attributable to the benefits they offer. However, this is not the case in Uganda, where there is a paucity of knowledge regarding the critical factors that have led to the low and slow rate of adopting fruit tree farming as an alternative to farming systems in Uganda and the Lake Kyoga region in particular, despite the suitable ecological support. Some studies have attributed it to a lack of awareness of their food value, limited market, absence of seeds in local nurseries, insufficient information on propagation techniques, ambition, and other social and economic constraints [35–38].

Recent studies have demonstrated that the effects of climate change are being experienced in all regions of Uganda [39]. In the Lake Kyoga basin, where Budaka district is situated, climate change and variability have significantly impacted the area through changes in rainfall patterns, temperature increase, and more frequent extreme weather events like floods and droughts [40]. These changes have disrupted agriculture, which is a major economic activity in the area, and they have damaged infrastructure, leading to economic losses and social stagnation [11, 41, 42]. For the last 5 years, there has been a notable decline in mango harvests, a dominant fruit tree in the area. This has been attributed to the variability and change in rainfall patterns and seasons and generally high annual rainfall during the flowering periods. With approximately 40% of the population in Uganda engaged in subsistence farming [43], integrating fruit tree cultivation would enhance food security whilst simultaneously mitigating the effects of climate change. Nevertheless, the success of adopting fruit tree cultivation in farming systems is contingent upon the socioeconomic and environmental factors that pertain to the farmers in question.

Research has been done examining factors influencing tree planting and adoption amongst households. Versteeg et al. [44] examined factors for the adoption of small-family forest plantations, Dinh et al. [45] studied the economic incentives and factors for tree planting in central Vietnam, Jamala et al. [46] considered factors for agroforestry practices in Nigeria, and Jha et al. [47] also focused on agroforestry in Tanzania. Kaudo et al. [48] studied tree planting behaviours by people in their private spaces, whilst others like Gebru et al. [49], Khalwale et al. [50], Kakuru et al. [51], Kalanzi et al. [10], and Sebukyu & Mosango [52] all analysed factors for the adoption of tree planting. However, all these studies lack species-specific focus, hindering concrete conclusions about specific tree types [53], and none of the above studies have attempted to map spatial patterns of adoption. Fruit tree crops, in particular, offer efficient resource utilisation, leading to higher income whilst sustaining ecosystems [54].

Despite government initiatives like the Plan for Modernisation of Agriculture (PMA) and National Agriculture Advisory Services (NAADS) promoting commercial crop cultivation, fruit tree adoption in Budaka District remains low at 14% [55]. This contributes to Uganda's overall low agricultural productivity, which grows at a mere 2% annually [56]. Consequently, there is a significant knowledge gap concerning fruit tree distribution, adoption rates, and species diversity in Budaka. Whilst research has often focused on determinants of adoption, including sociodemographic, economic, and institutional factors (e.g., education, income, and access to extension services), there is a lack of studies that systematically link these determinants to actual adoption practices. Recent systematic analyses by Menghistu et al. [57] and Magesa et al. [58] highlight this disconnect, emphasising the dominance of logistic regression studies without empirical follow-up on real-world implementation and impacts. Moreover, little information exists on factors affecting fruit tree adoption as a climate change adaptation strategy, especially in Budaka District. This present study is the first of its kind to assess the determinants of fruit tree adoption, employing a combined approach incorporating GIS-based spatial mapping and socioeconomic analysis. To achieve this, the study was guided by the following objectives:

1. To describe the characteristics of fruit tree gardens amongst smallholder farmers in Budaka District;
2. To map their spatial distribution of free tree adopters;
3. To assess farmers' awareness and perceptions of climate change and its relation to fruit tree cultivation and,
4. To identify the socioeconomic factors influencing the adoption of fruit tree farming as a climate adaptation strategy.

## 2. Methods and Materials

### 2.1. Description of the Study Area

**Figure 1:**
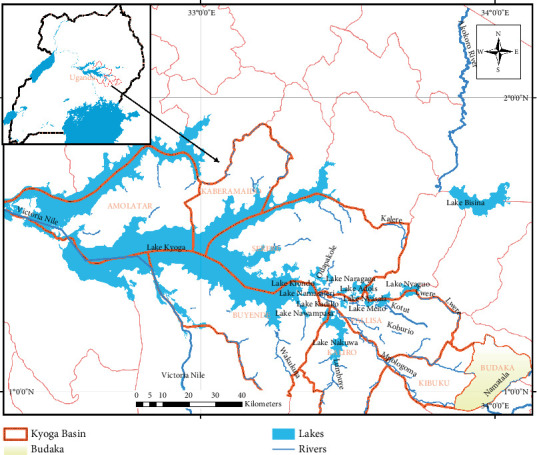
Location of the study area.

Budaka District is located at 1° 0⁣′ 14⁣^″^ North and 33° 54⁣′ 32⁣^″^ East, within the Lake Kyoga basin in eastern Uganda (Figure 1). The terrain is mostly flat, characterised by shallow seasonal wetlands. The elevation ranges from 900 to 1200 m above sea level, with an average of 1080 m. The area experiences two distinct rainy seasons: from March to June and from August to November, with an annual average rainfall of 1465 mm and an average temperature of 28°C, which varies monthly. The district is drained by two main rivers, Namatala and Lwere, which flow down the slopes of Mount Elgon through the plains in Budaka and eventually into the Mpologoma River and Lake Kyoga wetlands.

The soils here are ferralitic and hydromorphic, characterised by a reddish-brown colour and sandy loam texture, along with loams developed on laterite. These soils have a low pH of less than 5 and lack adequate phosphorus and exchangeable bases. They are suitable for growing crops such as sorghum, millet, groundnuts, cassava, pigeon peas, and cotton. Hydromorphic soils found in permanent or seasonal swamps and wetlands are often waterlogged, especially in the southern part of the district. Some of these soils show high levels of cation saturation, which may lead to localised saline conditions. These soils are ideal for cultivating paddy rice, sorghum, maize, and finger millet. The vegetation in the area has been heavily modified by tree cutting, grazing, and seasonal or biennial grass fires, often exacerbated by traditional farming practices. The dominant vegetation cover is savannah grassland. Swampy vegetation is common along the district's major wetlands. Isolated pockets of forest remain in the district. The study was specifically conducted in the four subcounties of Kakule, Iki-Iki, Lyama, and Nansanga.

### 2.2. Methods and Tools

**Figure 2:**
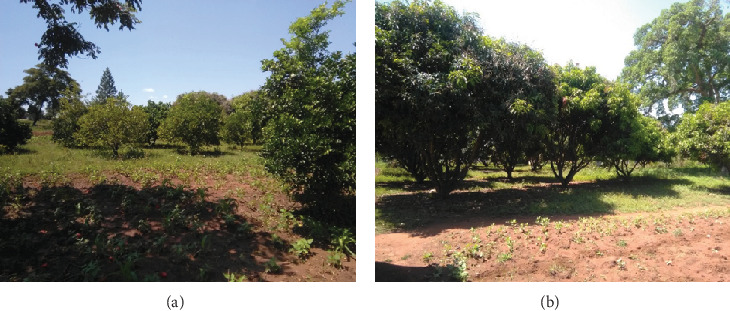
(a) Citrus and mangoes intercropped with beans/maize; (b) mangoes only intercropped with beans (this is a common farming system in the area).

This study employed a cross-sectional research design utilising a mixed methodology. This facilitated the collection of both quantitative and qualitative data from primary and secondary sources. This study design was considered appropriate for acquiring information on determinants of adoption at a specific time. Longitudinal data might offer more insights into the temporal dynamics and long-term considerations in adaptation, but such collection of data requires time and logistics, which were not available. Furthermore, panel data on smallholder agroforestry systems are often scarce in low-data settings such as Eastern Uganda. Nonetheless, cross-sectional designs have been effectively applied in comparable adoption studies to identify important relationships and trends [59]. The unit of analysis was the household. The study area was stratified into two ecological zones on the basis of the intensity of fruit growing. The data collection tools included the pretested questionnaires, observation checklists, interview guide, focus group discussion (FGD) guide, GPS (Trimble R1, 1–2 m accuracy) for plotting of coordinates, and a camera for taking photographs showing the current state of fruit tree gardens, such as in Figure 2. The data was collected using questionnaires directly administered to the farmers; interviews with key informants and technical staff at the district, subcounty, and parish; observations; documentary review; and FGDs.

### 2.3. Sample Selection

Whilst the Kyoga basin covers over 34 districts, Budaka district was conveniently selected due to its easy accessibility, that is, location along a major highway, and prior knowledge about the adverse effects of climate change and fruit farming in the study area was also considered in the selection. Budaka has 14 subcounties and 6 town councils, which were stratified based on the observable number of fruit farms into high-density and low-density growing areas. Four subcounties were randomly selected from the two strata/ecological zones, choosing two from each zone. These include Iki-Iki and Kakule subcounties for high-density fruit growing and Nansanga and Lyama from low-density fruit farming strata. Two parishes were randomly selected from each subcounty, and within the parishes, four villages were randomly selected, from which a total of 276 household heads were selected using systematic random sampling.

The sample size (heads of household) was determined using the Krejcie and Morgan [60] Table for determining the sample size. Based on a total population of 1500 households [61], the appropriate sample size at a 95% confidence level and 5% margin of error is 306. A slightly smaller sample of 276 households was considered due to logistical and time limitations. In addition to the households, we also interacted with key informants, including the subcounty community development officer, the agricultural extension officers, and chairpersons of the local councils. The sampling frame utilised the village registers for easy identification of potential respondents. Informed consent was obtained from all participants before data collection, and the respondents were informed about the purpose of the study and assured of the confidentiality of their responses. Ethical approval was also obtained from the Research Ethics Committee of Kyambogo University (KyuREC28/03/2024). The sampling procedure faced limitations, including participant availability and reluctance to share information.

### 2.4. Data Analysis

The data were analysed using descriptive statistics (frequencies and percentages), cross-tabulation tables, and chi-square (*x*^2^). The chi-square analysis was selected since the data has a dependent variable (dichotomous adoption and no adoption) and some independent variables, like gender. The aim was to establish whether there were any significant differences in socioeconomic characteristics between adopting and nonadopting farmers. The results are used to predict the likelihood of adopting or not adopting. Thematic and content analysis was used to analyse FGD, observation, and key informant data to identify key themes related to fruit tree adoption and climate change adaptation. GIS software (ArcMap 10.7) was used for map visualisation.

## 3. Results

### 3.1. Characteristics of the Fruit Trees and Gardens

The characteristics of fruit trees grown in the area and gardens that were covered in this study included the farms, the types of fruits grown, the form of growing the fruits (mixed or not), farm size (acreage), the number of fruits per farm, and the general distribution of fruit growing spatially in the study area. The types of fruits grown in this area (Table 1) are mostly early-maturing, disease- and drought-resistant varieties of mangoes, citrus, jackfruits, and avocados. Fruits are grown in a mixed system, where many farmers are found to be growing more than one variety. Other farmers intercrop fruit trees with food crops like beans, millet, sorghum, and other cereals (Figure 2). The most dominant combinations are mangoes and citrus (38%), mangoes, citrus, and avocado (36%), mangoes only (15%), and mangoes–citrus–jackfruits (11%). Mangoes occupy a larger acreage in this area than other fruit trees.

Concerning farm size and number of fruits grown by farmers in the study area, it was found that the majority of the farmers had less than one acre of land on which fruit trees were planted. Many farmers had fruit trees in their backyards. The average number of trees in the gardens varied from 18 to 60. This can be observed in Table 2.

**Figure 3:**
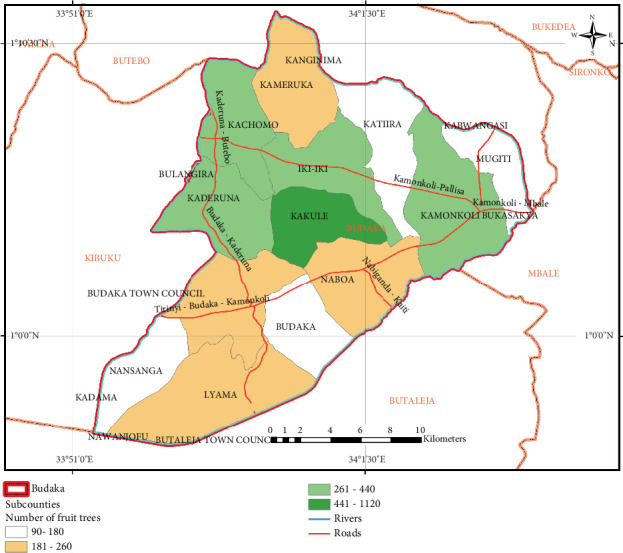
Spatial distribution of fruit trees in the study area (software: ArcMap 10.7; coordinate system: WGS 1984).

The density of fruit tree farms in the area was analysed using GPS plotting, where the number of fruit farms was enumerated and visualised using ArcMap. The spatial distribution of fruit tree farms is presented in Figure 3. The intensity increases as you move from the southwest to the northeast of the district. Kakule subcounty is the highest growing area based on the number of fruit farmers compared to other subcounties in the district. Kakule subcounty exhibits a marked geographical concentration of fruit tree adopters, characterised by a significantly higher density of fruit tree farms compared to other subcounties in the district. This clustering indicates that localised ecological compatibility, institutional support, and farmer-to-farmer dissemination may all be influencing adoption trends. Consequently, Kakule emerges as a geographical hot spot for fruit tree cultivation in Budaka district, signifying its importance for targeted future research and policy.

### 3.2. Awareness of Climate Change and the Farmers' Perception of Fruit Trees as an Adaptation Strategy in the Study Area

Generally, there is enough literature that indicates that farmers have reasonable awareness about climate change, although understanding the specific causes and potential impacts is still limited. Most farmers are aware of shifting weather patterns and their impact on farming [62]. However, many lack information about the specific causes and how certain farming practices can serve as adaptation measures and also provide a livelihood. In this study, we interrogated the fruit tree farmers about their awareness concerning climate change, causes of climate change, and their perception towards fruit tree growing as crops and climate change adaptation and mitigation, and the results are presented in Table 3.

It can be observed from Table 3 that the majority of the farmers in the study area (66%) are aware that farmers are responsible for climate change through poor land management practices. However, during a FGD, they indicated that industrial development and general urbanisation also contribute to climate change through land clearance to create space for industrial establishments. The highest disagreement is that natural factors contribute to climate change. They also agree that poor land management practices and forest clearance contribute to climate change.

Farmers also indicated that they are aware of climate change elements: increasing temperatures in the area, especially at night (87%); unreliable and unpredictable rainfall (55%); longer dry seasons (80%); and increasing occurrences of floods. Other signs of a changing climate that farmers indicated during the FGDs are invasive vegetation species, short growing seasons, and increased incidences of pests. When asked during FGDs whether they were adopting fruit tree farming as an adaptation strategy, 42% agreed, 20% were not sure, and 38% disagreed.

It was reported that many farmers had adopted fruit tree growing primarily for their livelihood, in line with the campaign championed by leaders, as a poverty alleviation measure amongst rural households. Selling the harvest can provide food security at the household level. The perception of farmers towards fruit tree growing as an adaptation strategy in Table 4 indicates that the majority of farmers currently perceive fruit growing as a climate change adaptation strategy. They noted that fruit growing improves local environmental quality, especially by cooling the air during the dry season, when temperatures are generally high (92%). During the FGD, they indicated that homesteads with fruit trees, especially mango trees in their backyards, experience cooler weather compared to those without fruit trees. Farmers also grow vegetables under tree shade during the dry season.

From Table 4, p. 10, we observe that 70% of farmers agree that fruit trees absorb carbon from the atmosphere and produce fresh oxygen, 74% agree that trees contribute to soil fertility in gardens, 92% reported that fruit trees provide evergreen shade year-round, 55% acknowledge the role of fruit trees in enhancing water infiltration into the soil, and 90% agreed that fruit trees are important for the supply of fuelwood, which has lessened pressure on forest clearing and environmental degradation. However, during the FGD, it was noted that climate change, which has affected seasonal weather patterns, has impacted the productivity of fruit trees, especially mangoes. For instance, the past 3 years have seen a decline in mango and citrus production because the flowering season now coincides with the wet season, negatively affecting pollination. Many farmers have started selling their fruit trees for wood energy used in brick firing, charcoal production, and domestic firewood. Additionally, increased pest and disease outbreaks have worsened the situation.

### 3.3. Socioeconomic Determinants of Fruit Tree Adoption in the Study Area

The socioeconomic determinants for the adoption of fruit tree farming in the area include age, education, gender, marital status, family size, type of labour employed on the farm, income, access to credit, and access to extension services. It was hypothesised that these factors significantly influence the adoption of fruit tree farming. The chi-square results are presented in Table 5. According to the descriptive statistics, over 60% of the farmers were above 40 years old and 2.8% were 18 years old or below. Generally, old farmers dominate the business of fruit tree growing. It was also established that about 70% of the fruit tree farms are owned by men. There are issues of gender imbalance in this sector. Most of the farmers had no education or had only completed primary (71%). The majority of fruit tree farmers reported an average family size of 12. This was generally higher than the national average, which is estimated at 5 [61]. Over 70% of fruit farmers in this area rely on family labour. In relation to farm size, the majority of the farmers had between 1 and 2 acres of land devoted to fruit tree growing. Many kept fruit trees on small pieces, especially near the homesteads. Generally, incomes of fruit tree farmers were higher than those who were not growing fruit. Their average income in a year was 2 million, which is about $600. The majority of fruit tree farmers reported not receiving credit from financial institutions (70%). In relation to extension services, it was found that the majority of the farmers have never received extension support despite the political support behind fruit farming.

### 3.4. Difference Between Adopters and Nonadopters of Fruit Tree Growing, and their Socioeconomic Characteristics

A chi-square analysis (*x*^2^ test) was used to establish whether there are significant differences between adopting and nonadopting farmers in terms of their socioeconomic characteristics and to ascertain the significant and less significant determinants of adoption. The results of the analysis are presented in Table 5.

The results in Table 5 indicate that out of the 10 factors considered in this study, only five indicated significant differences between adopters and nonadopters of fruit tree farming in this area. These included age of the farmers, type of labour used on the farms (hired), farm size, number of fruit trees planted by the farmers, and income of the farmers, both from the farm and off-farm activities. These findings suggest that these variables significantly influence the adoption of fruit tree farming. The other factors did not indicate statistically significant differences between adopters of fruit trees and nonadopters. Therefore, they are not likely to influence farmers to adopt fruit tree farming.

## 4. Discussion

### 4.1. Characteristics of the Fruit Farms in the Study Area

The most dominant fruit tree species grown in Budaka district are mangoes, citrus, avocados, and jackfruits. This is due to the region's tropical climate with distinct wet and dry seasons, which favour the flowering and yielding of mango trees [63–65]. Whilst traditional varieties existed, government and NGO initiatives introduced high-yielding, early-maturing cultivars like *Kakule*, *Boribo*, and Apple Mango. The political campaigns aimed at poverty alleviation have contributed to the adoption of mangoes as the leading species. Also responsible for the increase in production is the ready market along the Mbale-Tirinyi highway, where several roadside markets are common. Hence, mangoes and other fruits grown in this area provide economic opportunities, and they also enhance food security [66, 67]. Unlike other areas in Uganda, such as the central and western regions, bananas, pineapples, and passion fruits are more common because of the differences in altitude, rainfall, and soil types [10, 52]. Budaka is well known for fruit trees, including mangoes, which highlights a unique regional adaptation. For example, in Masaka and Mityana districts (Central Uganda), agroforestry interventions have concentrated more on integrating wood and fodder trees alongside bananas [52], whereas in the western highlands, passion fruits and apples are being pushed owing to the colder climate [10].

Many farmers in Budaka district still grow fruit trees on a small-holding basis, given that the average farm size was a mere one acre. Backyard gardens are the most dominant, and mixed cropping is common. This is attributable to land fragmentation resulting from the rising population in the area [26]. This leaves farmers with small parcels of land insufficient to cater for both food crop growing and fruit trees. Therefore, mixed cropping needs to be emphasised if adoption rates are to increase, particularly for tree species like mango that offer both nutritional and income benefits.

The spatial distribution of fruit farm trees depicts a discernible pattern that is attributable to drainage, land tenure systems, and other socioeconomic factors. Iki-Iki and Kakule subcounties dominate fruit tree growing due to good soils that support fruit trees [50], compared to Lyama and Nansanga, characterised by swamps and poorly drained soils. The existence of the swamps implies that the farmers choose rice growing over fruit tree farming. Hence, adoption is done as livelihood diversification. Worth noting is that Iki-Iki subcounty is home to the only district farm institute where some of the fruit varieties are developed. Little wonder, therefore, that areas closer to the farm institute (Kakule) have higher adoption rates compared to distant ones like Lyama and Nansanga. The observed spatial clustering of adopters in Kakule subcounty points to how the accessibility to support institutions, conducive physical environment, and social interaction might influence fruit tree adoption. Such clusters serve as demonstration areas and entry points for broader regional adoption of fruit trees.

### 4.2. Awareness and Perceptions of Farmers About Climate Change, and Growing Fruit Trees as an Adaptation and Mitigation Strategy

Whilst the majority of the farmers in the study area acknowledge being aware of climate change and its causes, they also agree that human activities have contributed to this phenomenon. For this, most farmers who have adopted fruit tree growing now perceive it as an adaptation strategy both in terms of environmental modification and provision of alternative livelihood. With this new awareness, efforts are needed to encourage more farmers to join fruit growing. Changing the narrative that fruit trees are not merely sources of food and income but also contribute to the atmospheric moisture and formation of convection rainfall is important, given the fact that many farmers may not be willing to abandon food crop growing for fruit tree planting. The desire to meet household livelihood also makes farmers undertake decisions like poor management of land and forest clearance, despite being aware that this affects the environment [62, 68–70].

There is a mismatch between farmers' awareness of climate change effects and their willingness to adopt fruit trees as an adaptation strategy. It was found out that whilst the farmers are aware of the climate change phenomenon, many have not embraced agroforestry as an adaptation strategy. This discrepancy is partly attributable to behavioural economic theories such as risk aversion and prospect theory [71]. Smallholder farmers operate under uncertainty and incur high opportunity costs when they invest in long-term ventures like fruit tree farming. The lagged economic benefits, exposure to climate variability, pest invasion and other competing short-term livelihood priorities make such choices risky even if long-term gains are familiar. Adoption is thus not just a matter of knowledge but also of subjective risk perception, access to resources, and institutional support [72]. Livelihood diversification, where people do not have to rely on the land alone as a source of livelihood, would help realise the benefits of fruit trees in mitigating the effects of climate change. As noted by Dinh et al. [45], Wakaba et al. [73] & Zaca et al. [31], combating the effects of climate change requires concerted efforts that are holistic, inclusive, and gender sensitive.

### 4.3. Socioeconomic Factors Influencing the Fruit Tree Adoption in the Study Area

#### 4.3.1. Gender and Age Dynamics

The study revealed a persistent gender gap in the agricultural sector, consistent with broader trends in developing countries [74–76]. Whilst women actively participate in farm labour, the customary land tenure system does not permit women to own land, and they often lack decision-making power. This partly explains why gender did not significantly influence fruit tree adoption despite women's visible role in farming [47]. Addressing the gender gap in land ownership is crucial for improving adoption rates, as women would then have the authority to make decisions. This aligns with research by Akudugu et al. [77] and Badstue et al. [78], who found that gender disparities in land ownership were major obstacles to adopting climate change adaptation strategies. Fasakin et al. [79] also note that the lack of land ownership rights limited youth participation in agriculture amongst rural households in Nigeria.

Similarly, age structure significantly affects fruit tree adoption since land is owned by older individuals who have the power to decide land use. Farmers aged 40 and above were significantly more likely to own orchards than their young counterparts. Although there is a decline in agricultural interest amongst the younger population [80], young people lack land ownership rights needed to make such decisions. Many rely on rented land for their livelihood and are hesitant to grow perennial crops like fruit trees due to short-term tenancy [81]. Conversely, older farmers dominate the sector, likely because of their experience and ability to manage challenges. These findings align with Esabu & Ngwenya [82], Basamba et al. [83], and Permadi et al. [84] but contrast with studies showing a negative correlation between age and adoption [85–88]. This contrast could be attributable to differences in cultural and tenure systems. It is possible that extended family farming systems, more prevalent in parts of West and Central Africa, may offer younger farmers greater informal access to land and involvement in decision-making, in contrast to more individualised tenure systems observed in Uganda. Similarly, Arwida et al. [87] found that customary tenure in Kalimantan, Indonesia, allowed young farmers to claim land ownership through lineage, hence gaining access without formal documents.

#### 4.3.2. Labour, Landholding, and Income

Labour availability, that is, dependence on family labour, was a good predictor of adoption. For smallholder production systems where rented labour is either expensive or unreliable, the greater the number of family members in a household, the more likely it is to plant and retain fruit trees [13]. Land size also positively affected it, in line with findings indicating that farmers with broader land expanses are more likely to diversify into perennials [51, 81, 84, 89, 90]. This finding also contrasts with Biresaw et al. [9] and Quezada et al. [34], who reported a negative relationship in a different context. Land availability is a key determinant of adoption, whereby mostly farmers with large chunks of land can devote land for orchards [91].

Land availability, indicated by the number of fruit trees planted, was found to be a significant determinant of adoption. Farmers with large chunks of land can devote land for fruit tree growing in addition to other land use activities. Relatedly, wealthier households are also more likely to own large acreages of land, possess the financial resources required for inputs and investments, and await delayed returns from fruit trees. This agrees with Syano et al. [92], Kakuru et al. [51], and Dinh et al. [45], who emphasise the role of disposable income on long-term agricultural investment.

#### 4.3.3. Education, Access to Credit, and Extension Services

The levels of education amongst the farmers did not significantly influence the adoption of fruit trees in Budaka district, revealing a contrast with previous studies [83]. This could be due to the overall low levels of education in Budaka District, the prevalence of informal knowledge systems, and a potential bias in the sample population. Rural–urban migration amongst the educated people is common in this part of Uganda, leaving behind only the older, uneducated population. However, these findings are supported by previous research [9, 81, 92–96], which noted that education did not significantly influence adoption. This suggests that factors beyond formal education may drive adoption decisions in this context.

Access to credit did not differ much between the adopters and nonadopters of fruit trees. This could be attributable to the limited formal financial institutions, leaving the farmers to rely on informal credit sources. Informal credit is usually associated with unfair lending terms, and banks are typically reluctant to lend to farmers given the high risks and uncertainty in the agriculture sector [83]. Simultaneously, little contact with extension officers reflects a great limitation in the agricultural support system amidst concerted efforts from the government. Extension contact helps to build perception and attitude amongst the farmers and must be addressed to enhance fruit tree farming activities [77].

### 4.4. Policy Implications

The findings of this study highlight several policy interventions that could be implemented to encourage the adoption of fruit trees as a strategy for adapting to climate change. In the short term, the most viable and cost-effective intervention is to expand inclusive agroforestry extension services. This could be achieved by leveraging existing district-level infrastructure, such as DATIC, thereby reducing the additional institutional burden. In the medium term, improving smallholder farmers' access to credit, particularly through collaboration with rural financial institutions and savings cooperatives, is essential. To make lending to farmers viable, such interventions must be coordinated, and financial literacy programs implemented alongside agricultural risk insurance investments. Reforming land tenure is a crucial but long-term priority, especially to protect the property rights of women and young people. These reforms are complicated, involving institutional constraints and customary law, and requiring both community awareness-raising and national-level legal action. However, the long-term adoption of fruit tree systems will continue to be limited unless tenure insecurity is addressed, particularly for women and young people. Finally, geographic hotspots with high adoption rates, such as Kakule subcounty, should prioritise pilot interventions. Through community-based platforms and peer learning, these can serve as demonstration hubs, accelerating diffusion.

### 4.5. Limitations of the Study

The present study encountered certain limitations. The utilisation of self-reported data is prone to recall bias and social desirability bias arising from respondents overestimating or underestimating their fruit tree adoption practices. Furthermore, the choice of Budaka district was by convenience and data availability, and this could hamper the generalisation of the results to other regions with distinct agroecological and institutional settings. The use of the cross-sectional study design provides a single snapshot of the adoption behaviour at a specific time. This constrains the ability to study the temporal dynamics of adoption and understand how it evolves with time. These limitations could be mitigated through future research that expands the study design to include longitudinal panel data, covers larger geographical areas encompassing several districts, and incorporates remote sensing techniques to corroborate self-reported data.

## 5. Conclusions

The study investigated the socioeconomic and spatial determinants of fruit tree adoption in Budaka district. The first objective aimed to describe the characteristics of fruit tree gardens. This was addressed using household survey data, which revealed that fruit tree farming in Budaka district is mainly practised on smallholder farms, mixed-use plots where fruits are intercropped with other seasonal crops. Many farmers also own backyard gardens where fruits are grown as a source of livelihood. A GIS-based analysis revealed that Kakule and Iki-Iki subcounties have the highest adoption levels, forming a spatial cluster of fruit tree adopters probably driven by the close proximity to institutional support centres like the District Agricultural Training Institute (DATIC), active farmer networks, favourable soils, and political campaigns. Government policy, land availability, age, access to credit, and gender are the significant factors influencing the adoption of fruit tree farming. Very notable gender disparities exist in the ownership of fruit tree farms, and young farmers need land ownership rights to decide whether to adopt or not.

Although many farmers are aware of climate change and the role of fruit trees in mitigating climate change effects, there still exist gaps in the perception amongst farmers about fruit tree growing as a climate change adaptation strategy, driven by economic conditions and land tenure issues. This information is important to inform government policy on the promotion of agroforestry. Strengthening local institutions like DATIC and implementing inclusive land policies should be undertaken as critical steps to scaling agroforestry in climate-vulnerable regions. Future studies should focus on exploring long-term fruit yield trends under changing rainfall regimes and test pilot-scale incentive models. Furthermore, in order to identify causal pathways in adoption behaviour and more accurately evaluate the sustainability of agroforestry practices over time, future research should take into account experimental or longitudinal designs. This study provides a novel approach that combines spatial GIS-based mapping with socioeconomic analysis of the determinants of fruit tree adoption. The study offers a unique perspective on fruit trees as a strategy for adapting to climate change in Eastern Uganda and provides a foundation for geographically focused policy interventions by connecting spatial patterns to adoption behaviour at household level.

## Figures and Tables

**Table 1:** Fruit trees grown on the farms.

**Categories of fruits**	**Percentage**
Mangoes + citrus + avocado	36%
Mangoes + citrus	38%
Mangoes + citrus + jackfruit	11%
Mangoes only	15%

*Note: n* = 276.

**Table 2:** Average number of fruit tree farms in the study area.

**Size of fruit farm (acres)**	**No. of farmers**	**Average number of fruit trees**	**% of farmers**
1 or fewer	131	18	56
2	78	38	33
3	18	50	08
4	08	60	03
Total	276		100

*Note:* Source: field data.

**Table 3:** Awareness of causes of climate change and variability in the area (percent).

**Causes**	**Strongly disagree**	**Disagree**	**Not sure**	**Agree**	**Strongly agree**
Farmers are responsible	5	8	21	47	19
Natural factors	20	48	5	2	25
Poor land management	3	7	20	51	16
Forest clearing	4	5	20	13	16

**Table 4:** Farmers' perception of fruit trees as climate change adaptation (percent).

**Perceived role**	**Strongly disagree**	**Disagree**	**Not sure**	**Agree**	**Strongly agree**
Improve on the environment	—	—	8	22	70
Absorb carbon	5	6	25	20	50
Improve fertility	6	8	22	34	40
Evergreen	0	0	08	32	60
Improve infiltration	10	15	20	20	35
Provide firewood	—	—	10	40	50

**Table 5:** Statistical analysis (*x*^2^ test) to determine whether there are significant differences in socioeconomic factors between adopters and nonadopters of fruit tree growing.

**Variable**	**Chi-square (** **χ** ^2^ **) result**	**p** ** value**	**Significant**
Age	*χ* ^2^ = 22.495, df = 3	*p* < 0.001	Yes
Gender	*χ* ^2^ = 3.434, df = 1	*p* = 0.064	No
Education	*χ* ^2^ = 2.792, df = 3	*p* = 0.425	No
Family size	*χ* ^2^ = 0.286, df = 2	*p* = 0.867	No
Type of labour	*χ* ^2^ = 160.479, df = 3	*p* = 0.001	Yes
Farm size	*χ* ^2^ = 138.988, df = 4	*p* < 0.001	Yes
Income	*χ* ^2^ = 133.657, df = 5	*p* < 0.001	Yes
Access to credit	*χ* ^2^ = 3.692, df = 1	*p* = 0.055	No (marginal)
Number of fruit trees planted	*χ* ^2^ = 4.456, df = 1	*p* = 0.030	Yes
Extension services	*χ* ^2^ = 3.477, df = 1	*p* = 0.062	No

## Data Availability

The corresponding author can provide access to the data upon reasonable request.
